# Community Transmission of Extended-Spectrum β-Lactamase

**DOI:** 10.3201/eid0908.030094

**Published:** 2003-08

**Authors:** Beatriz Mirelis, Ferran Navarro, Elisenda Miró, Raul Jesús Mesa, Pere Coll, Guillem Prats

**Affiliations:** * Hospital de la Santa Creu i Sant Pau, Barcelona, Spain; † Universitat Autònoma de Barcelona, Barcelona, Spain

**To the Editor:** The spread of multiresistant gram-negative bacteria in the general population is a problem of paramount importance, but the responsible mechanisms are poorly understood. Several studies have focused on β-lactam resistance in *Enterobacteriaceae* isolated from stools in healthy people, but they did not specifically investigate the extended-spectrum β-lactamases (ESBL). Furthermore, none of these studies detected ESBL in the evaluated population (*1*,*2*). We performed three survey studies to determine the incidence of *Enterobacteriaceae* strains producing ESBLs in the stools of outpatients attending our hospital. The first study was performed during a 4-month period (February–May 2001), the second during a 3 month-period (April–June 2002), and the third during 1 month (October 2002).

Stool samples were spread onto plates of MacConkey agar containing 2 mg/L of cefotaxime. A colony of each distinct morphotype was analyzed further. Species were identified according to conventional methods (*3*). The susceptibility to β-lactam antibiotics was determined by the disk-diffusion test, following recommendations of the National Committee for Clinical Laboratory Standards (*4*,*5*). The interpretative reading of the antibiogram was performed according to standard guidelines (*4*–*6*). The MICs of cefotaxime and ceftazidime, with and without clavulanic acid, were later determined by E test (AB Biodisk, Solna, Sweden). Strains producing ESBL were defined as strains showing synergism between amoxicillin-clavulanic acid and cefotaxime, ceftazidime, cefepime, or aztreonam (*4*,*5*).

All strains suspected of carrying a resistance pattern compatible with hyperproduction of the chromosomal enzymes, as well as resistant strains without synergy, were disregarded. During the first period, 15 (2.1%) of 707 outpatients were carriers of *Escherichia coli* (14 patients) or *Proteus mirabilis* (1 patient) with ESBL. This percentage increased during the second period, when 17 (3.8%) of 454 outpatients were carriers of *E. coli* with ESBL, and again in the third period, when 12 (7.5%) of 160 were carriers of *E. coli* (11 patients) or *Enterobacter cloacae* (1 patient) with ESBL. Characterization of the different ESBL isolated during the three study periods is in process. Although *Klebsiella pneumoniae* carrying ESBL has been detected in our hospital (*7*), as well as in other hospitals in Barcelona (*8*), no ESBL-producing *K. pneumoniae* strains were identified in this survey.

Although we did not disregard either the patients’ previous treatment with antibiotics or previous hospitalization, these patients came to the hospital from the community carrying strains that express ESBL. Moreover, during these three periods we observed a significant increase in the frequency of ESBL carriers (from 2.1% to 7.5%; p<0.005). These data suggest that the community could be a reservoir for these enzymes, as occurs with other microorganisms (*9*–*11*). Many questions remain unanswered regarding the diffusion mechanisms of this resistance in the community. Confirmation of community-based transmission of ESBL would indicate a need for heightened vigilance and further studies to determine the reservoirs and vehicles for dissemination of ESBL within the community.
